# Decrease in the Sensitivity of Myocardium to M3 Muscarinic Receptor Stimulation during Postnatal Ontogenisis

**Published:** 2016

**Authors:** S.V. Tapilina, D.V. Abramochkin

**Affiliations:** Department of human and animal physiology, Lomonosov Moscow State University, Leninskie Gory 1 bldg. 12,119234,Moscow, Russia; Department of physiology, Pirogov Russian National Research Medical University, Ministry of Healthcare of the Russian Federation, Ostrovityanova str. 1, 117997, Moscow, Russia

**Keywords:** acetylcholine, muscarinic receptors, heart, action potential, ontogenesis

## Abstract

Type 3 muscarinic receptors (M3 receptors) participate in the mediation of cholinergic effects in mammalian myocardium, along with M2 receptors. However, myocardium of adult mammals demonstrates only modest electrophysiological effects in response to selective stimulation of M3 receptors which are hardly comparable to the effects produced by M2 stimulation. In the present study, the effects of selective M3 stimulation induced by application of the muscarinic agonist pilocarpine (10 μM) in the presence of the selective M2 blocker methoctramine (100 nM) on the action potential (AP) waveform were investigated in isolated atrial and ventricular preparations from newborn and 3-week-old rats and compared to those in preparations from adult rats. In the atrial myocardium, stimulation of M3 receptors produced a comparable reduction of AP duration in newborn and adult rats, while in 3-week-old rats the effect was negligible. In ventricular myocardial preparations from newborn rats, the effect of M3 stimulation was more than 3 times stronger compared to that from adult rats, while preparations from 3-week old rats demonstrated no definite effect, similarly to atrial preparations. In all studied types of cardiac preparations, the effects of M3 stimulation were eliminated by the selective M3 antagonist 4-DAMP (10 nM). The results of RT-PCR show that the amount of product of the M3 receptor gene decreases with the maturation of animals both in atrial and ventricular myocardium. We concluded that the contribution of M3 receptors to the mediation of cardiac cholinergic responses decreases during postnatal ontogenesis. These age-related changes may be associated with downregulation of M3 receptor gene expression.

## INTRODUCTION

Parasympathetic regulation of the heart is extremely important for its proper functioning. The neurotransmitter acetylcholine (ACh) secreted by intramural postganglionic parasympathetic nerve endings is a major effector of the parasympathetic nervous system. ACh affects pacemaker and working cardiomyocytes through type 2 muscarinic receptors (M2 receptors), causing negative chronotropic and inotropic effects, respectively [1]. However, there is plenty of recent evidence of the existence of functionally active type 3 acetylcholine receptors (M3 receptors) in the mammalian myocardium [2-4].

While M2 receptors are coupled with G_i_ proteins and the main effects of their stimulation are associated with a decrease in the intracellular levels of cAMP, M3 receptors are coupled with G_q_ proteins, and, therefore, their stimulation results in the activation of the intracellular phosphoinositide signaling cascade [1, 2]. In this process, the α-subunit of the Gq protein activates phospholipase C, which ultimately leads to an increased intracellular level of Ca^2+^ and activation of protein kinase C capable of affecting the functioning of various ion channels by phosphorylation. On the other hand, the channels carrying the potassium current (I_KM3_) are apparently activated by direct interaction with Gq protein subunits [3, 5]. Stimulation of M3 receptors leads to a decrease in AP duration, which is mainly observed in atrial myocardium of adult rats [6], mice [4], and guinea pigs [7]. Furthermore, M3 receptors mediate a number of ACh effects that are not related to electrical activity; in particular its antiapoptotic effect on cardiomyocytes [8, 9].

Most research dealing with myocardial M3 receptors are limited to the study of their functions in adult animals, despite the fact that at the early stages of postnatal ontogeny, the role of parasympathetic cardiac regulation is generally higher than in adults due to underdevelopment or lack of sympathetic innervation of myocardium [10]. The results of *in vivo *experiments on infant rats [11], as well as preliminary results obtained by our group [12] for myocardium of newborn rats, suggest a higher sensitivity of myocardium to M3 receptor stimulation at the early stages of ontogeny.

In this regard, the present work included a comparative study of the electrophysiological effects of selective stimulation of M3 receptors in the atrial and ventricular myocardium of newborn rats (NRs) on the first day of life, three-week-old rats (TWRs), and adult rats aged 4 months (ARs). Electrophysiological data were compared to the expression of the M2 and M3 receptor genes measured by real-time PCR (RT-PCR).

## EXPERIMENTAL

We used four-month-old male albino rats (n = 26) weighing 300–350 g, TWRs weighing 24–28 g (n = 24, five different litters), and NRs weighing 4.5–6 g (n = 25). The animals were decapitated, the thorax was quickly opened, and the heart was isolated and washed with Tyrode’s solution (composition in mmol/l: NaCl 133.47; KCl 4.69; NaH_2_PO_4_•H_2_O 1.35; NaHCO_3_ 16.31; MgSO_4_•H_2_O 1.18; CaCl_2_•2H_2_O 2.5; glucose 7.77), saturated with carbogen (gas mixture of 95% O2 and 5% CO_2_). Then, a preparation of the right atrial appendage and a preparation of the right ventricular wall were isolated from each heart. Each preparation was mounted on a 3-ml experimental chamber (temperature 38°C, flow rate 10 ml/min) with its endocardial surface upward and stimulated using silver electrodes with a frequency of 6 Hz (ARs and TWRs) or 4 Hz (NRs).

AP was recorded using a standard method of intracellular recording of bioelectric activity with 25–50 MOhm glass microelectrodes connected to a Neuroprobe- 1600 amplifier (AM-Systems, USA). The signal was digitized using a E14-140 analog-to-digital converter (L-Card, Russia) and recorded on a computer using the Powergraph 3.3 software (DiSoft, Russia). Data processing was carried out using the MiniAnalysis v. 3.0.1 software (Synaptosoft, USA). When analyzing the records, we determined AP duration at 50 and 90% repolarization (APD50 and DPD90, respectively), as well as the AP amplitude and resting potential value.

In electrophysiological experiments, four compounds were used: selective blockers of the M1, M2, and M3 receptors; pirenzepine, methoctramine, and 4-DAMP, respectively; and the M receptor agonist pilocarpine, having low specificity to the M1 and M3 receptors as compared to the M2 and M4 receptors. All the substances were ordered from Sigma (USA). The concentrations of substances were selected based on data from previous studies [4, 7]. Each preparation was used no more than twice to record the pilocarpine effect under normal conditions and in the presence of a blocker.

Gene expression levels were compared by RT-PCR. Preparations of right atrial appendage and right ventricular walls from NRs, TWRs, and ARs obtained as described above were used for this purpose. The preparations were placed in a RNA stabilizing solution (IntactRNA, Evrogen, Russia) for 24 hours at 4C° and then stored at –20C° until RNA isolation. RNA was extracted using the guanidinium thiocyanate-phenol-chloroform method (ExtractRNA, Evrogen, Russia). RNA was purified from genomic DNA using DNase I (2000 act. units/ ml, NEB, USA) for 60 min at 37C°. The RNA concentration was measured using a spectrophotometer (Nanodrop 2000, ThermoScientific, USA). For cDNA synthesis, the resulting RNA purified from genomic DNA was subjected to a reverse transcription reaction using a MMLVRTkit kit (Evrogen, Russia). All manipulations were carried out in accordance with the standard procedures using the protocols recommended by the manufacturer. cDNA was stored at –80C° until RT-PCR.

RT-PCR was performed on a BioRad instrument equipped with a CFX96 detection system using a Synthol reagent kit (Russia) and EvaGreen dye (BIOTIUM, USA). We used primers synthesized at Evrogen (5’–’): M2 receptor –TCTACACTGTGATTGGTTACTGGC (forward), GCTTAACTGGGTAGGTCAGAGGT (reverse); M3-receptor –SAAGTGGTCTTCATTGCCTTCT (forward), GCCAGGCTTAAGAGGAAGTAGTT (reverse); GAPDH –CAGCGATGCTTTACTTTCTGAA (forward), GATGGCAACAATGTCCACTTT (reverse).

The amplification program consisted of initial denaturation at 95C°, 5min; followed by 50 cycles of PCR (1 min at 95C°, 30 sec at 60C°, and 30 sec at 72C°); and then the last step at 72C° for 10 min. Data were analyzed by the threshold method using the software supplied with the thermocycler. The results were normalized to the amount of RNA taken for the reverse transcription reaction.

The results were statistically processed using the Statistica 6.0 software. The Wilcoxon test was used to assess the statistical significance of the differences for paired samples; The Mann-Whitney test was used for unpaired samples. We used nonparametric tests due to the small sample sizes, which could not provide a normal distribution.

## RESULTS

Muscarinic receptor agonist pilocarpine (10 uM) was used for selective stimulation of M3 receptors in electrophysiological experiments. It was applied to the experimental chamber in the presence of the highly selective M2 receptor blocker methoctramine (100 nM). Special preliminary experiments, where pilocarpine was applied in the presence of the selective antagonist pirenzepine (100 nM), were used to eliminate a possible effect of M1 receptor activation. Since there were no differences in the intensity of pilocarpine effects in the presence of methoctramine and in the presence of two blockers, which is consistent with previous data showing the absence of M1 receptors in cardiomyocytes, pilocarpine was further applied in the presence of methoctramine alone for selective stimulation of M3 receptors.

In addition to registration of the M3 receptor stimulation effects, we conducted control experiments where pilocarpine was applied in the absence of blocking agents to assess the total effect of M2 and M3 receptor activation in myocardial preparations.

**Fig. 1:**
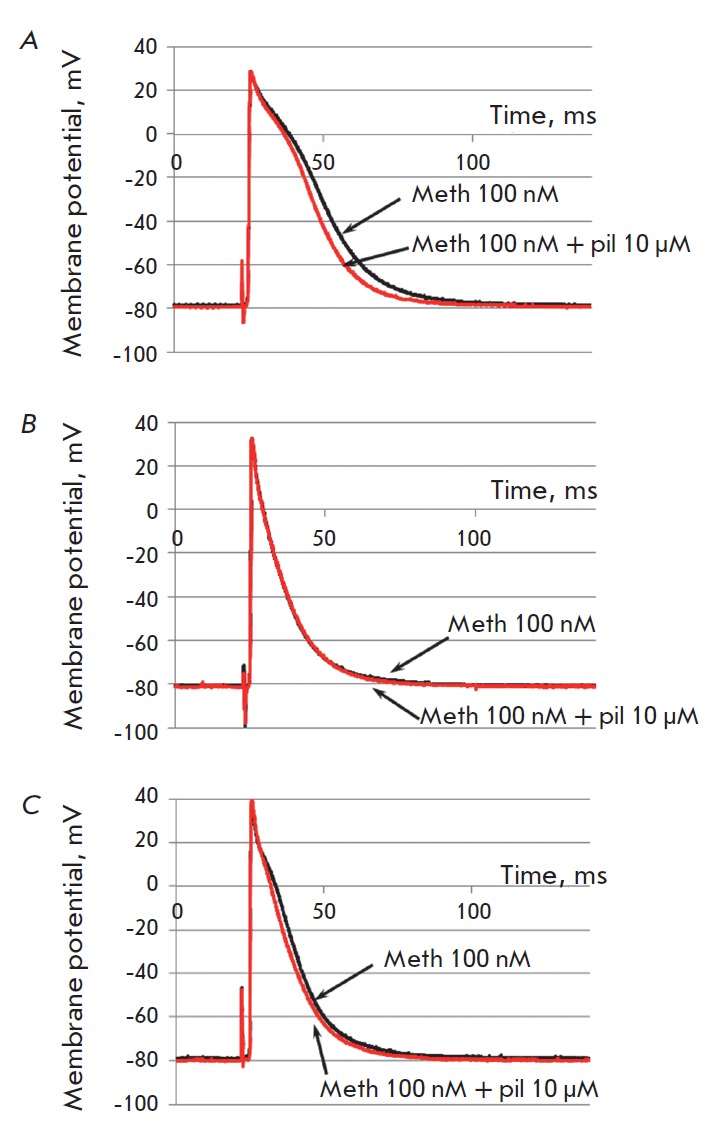
Comparison of original AP traces recorded in isolated right ventricular wall preparations of NR (A), TWR (B), and AR (C) in the control or during the maximum effect of pilocarpine (10 μM) in the presence of the selective M2 blocker methoctramine (100 nM).

**Fig. 2:**
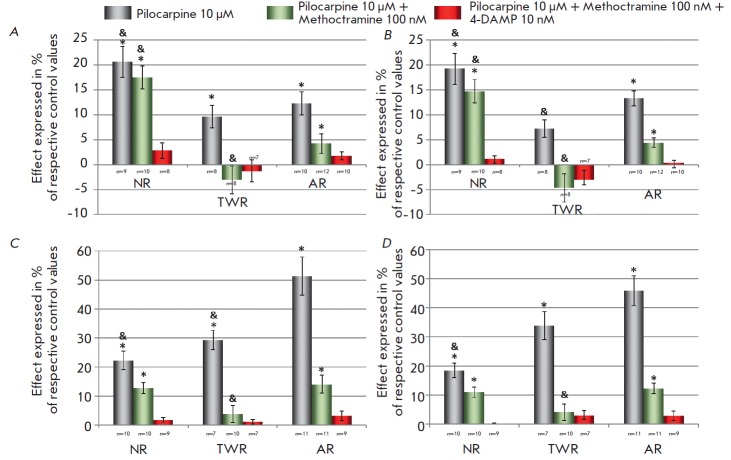
Relative reduction in AP duration in ventricular (A, B) and atrial (C, D) myocardial preparations measured at a 50% (A, C) and 90% (B, D) repolarization level induced by 10 μM pilocarpine under normal conditions or in the presence of methoctramine (100 nM) and 4-DAMP (10 nM). Ordinates: maximal pilocarpine-induced decrease in APD50 or APD90 expressed in % of respective control values. *p < 0.05 vs. the respective control values, Wilcoxon test. &p < 0.05 vs. respective effects in AR, Mann-Whitney test.

It was found that in the absence of blockers, pilocarpine significantly reduces AP duration both at 50% and 90% repolarization levels in the ventricular (*Fig. 1*, *Fig. 2A,B*) and atrial (*Fig. 2C,D*) rat myocardium in all three age groups. The maximum effect of pilocarpine developed within 250–300 s after the beginning of the application of the substance. Hereinafter, we will discuss only the maximum values of pilocarpine effects.

The effect of selective stimulation of M3 receptors in all series of experiments was qualitatively similar to the effect of pilocarpine in the absence of blockers, but it was significantly less pronounced. However, in the ARs and NRs, APD50 and APD90 were significantly reduced both in the ventricular (*Fig. 1A,C*, *Fig. 2A,B*) and atrial myocardium (*(Fig. 2C,D)*. On the contrary, there was no significant effect of selective stimulation of M3 receptors in the TWR group (*Fig. 1B*, *Fig. 2*). Almost no effects of the selective stimulation of M3 acetylcholine receptors were observed in the presence of 4-DAMP (10 nM), selective M3 receptor blocker; i.e., these effects were actually mediated by the activation of M3 receptors (*Fig. 2*).

It should be noted that the effect of M3 receptor stimulation in the ventricular myocardium of NRs was threefold stronger compared to that in ARs *(Fig. 2 A,B),* while no significant differences in the intensity of this effect were observed in the atrial myocardium. Thus, the most pronounced effect of M3 stimulation in the ventricular myocardium was observed for NRs, and the least pronounced effect was observed in TWRs. In the atrial myocardium, the main difference between the three age groups was observed in response to pilocarpine applied without blockers. The intensity of the effect increases with animal age, and it is more than twofold higher in ARs compared to NRs.

**Fig. 3:**
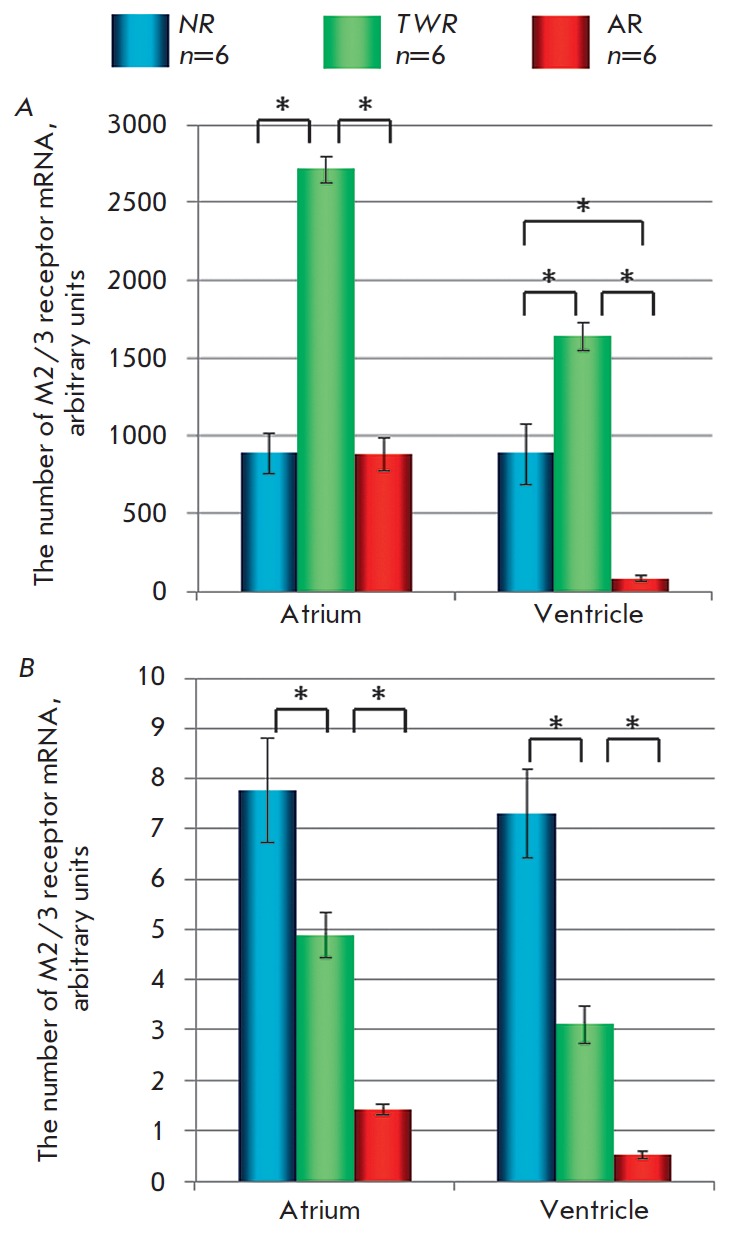
Expression level of the M2 (A) and M3(B) receptor genes in the atrial and ventricular myocardium of NR, TWR, and AR. Ordinates: arbitrary units of mRNA quantity. * - p < 0.05 – difference between two groups, Mann-Whitney test.

According to the results of RT-PCR, mRNA of both the M2 and M3 receptors is synthesized in the myocardium of animals of all age groups. However, the expression of the M3 receptor gene is much weaker *(Fig. 3).* Furthermore, the expression level of the M3 receptor gene decreases both in the atrial and ventricular myocardium with maturation of the animal *(Fig. 3B)*. Thus, it is higher in the myocardium of TWRs compared to that in ARs. However, the expression level of the M2 receptor gene was highest in the TWR group. Therefore, the ratio of M3 to M2 expression in the ventricular myocardium was higher in ARs compared to that in TWRs: 0.59 vs. 0.19%. In the atrial myocardium, the ratio was nearly the same: 0.16 and 0.18%, respectively.

## DISCUSSION

We were the first to obtain information on the change in the relative contribution of the M3 receptor to the regulation of the electrical activity of the ventricular and atrial myocardium during the postnatal ontogenesis of rats.

In electrophysiological experiments, selective stimulation of M3 receptors was achieved using a common method [4, 7]; more specifically, the application of 10 mM pilocarpine under conditions of total blockade of M2 receptors with 100 nM methoctramine. Please note that in our previous work, increase in the methoctramine concentration did not alter the pilocarpine effects, and, therefore, the effect observed in the presence of 100 nM of pilocarpine was unrelated to the activation of the residual M2 receptors. This fact is also confirmed by an almost complete elimination of the pilocarpine effect caused by both types of M receptor blockers: methoctramine and 4-DAMP.

Electrophysiological data suggest that the effect of M3 receptor stimulation on the electrical activity of the ventricular myocardium is maximal in NRs. In the atrial myocardium, sensitivity to pilocarpine in the absence of M receptor blockers increases with age, while sensitivity to pilocarpine under conditions of blockade of M2 receptors is identical in NRs and ARs. We can assume that the contribution of M2 receptors to electrical activity regulation increases with age both in atrial and ventricular myocardium, and in the ventricular myocardium of NRs M3 receptors play a key role.

The results of RT-PCR generally confirm these assumptions, since they show that expression of the M3 receptor gene decreases with age. It is still unclear why no effect of M3 receptor stimulation is observed in TWRs. On the one hand, this can be explained by the lowest ratio of M3 receptor mRNA to M2 receptor mRNA in this age group. On the other hand, the relative translational levels of M2 and M3 receptor proteins may differ from the expression levels of mRNA of the corresponding genes.

## CONCLUSION

In general, our results suggest an important functional role for the M3 receptor in the ventricles of newborn rats, which is leveled in ARs. Furthermore, M3 receptor functions are not limited to their action on the electrical activity investigated in our studies. For example, M3 receptors can participate in the realization of the cardioprotective effects of ACh [8, 9] under oxidative stress conditions experienced by a newborn’s body. It is unlikely that change in the role of the M3 receptor is related to the beginning of sympathetic regulation of the myocardium, since there is no effect of M3 receptor stimulation as early as at the age of three weeks, before sympathetic regulation is switched on.

## Abbreviations

ACh: acetylcholine
M2 receptors: type 2 muscarinic receptors
M3 receptors: type 3 muscarinic receptors
NR: newborn rats
TWR: 3-week old rats
AR: adult rats
RT-PCR: real-time PCR
AP: action potential
APD50: AP duration at 50% repolarization level
APD90: AP duration at 90% repolarization level
